# Supplementary material for: Mriyaviruses: Small Relatives of Giant Viruses
Source: bioRxiv. 2024 Feb 29:2024.02.29.582850. Preprint. [Version 1] doi: 10.1101/2024.02.29.582850 (PMC10962738; doi:10.1101/2024.02.29.582850)
Supplement: Supplement 1 [file media-1.pdf]

Supplementary figures

S1 Multiple sequence alignment (MSA) of *Yaraviridae* MCP proteins.

S2 MSA of “*Gamadviridae*” MCP proteins.

S3 Top hits of HHpred searches initiated with *Yaraviridae* and “*Gamadviridae*” MCP protein MSAs.

S4 VLTF2 protein MSA.

S5. Top hits of HHpred searches initiated with Mriyavirus VLTF2 protein MSA.

S6. Top HHpred hits for “*Gamadviridae*” (right) and *Yaraviridae* (left) RuvC MSAs.

S7. Phylogenetic tree of RuvC proteins.

S8. MSA of selected SF3_hel1 proteins.

S9. Phylogenetic tree of SF3_hel1 proteins.

S10. SF3_hel2 protein MSA.

S11. Top hits of HHpred search initiated with Mriyavirus HUH endonuclease MSA.

S12. MSA of HUH and C-terminal domain of HUH_long proteins.

S12. MSA of mriyavirus PDDEXK nuclease.

S13. Phylogenetic tree of PDDEXK nucleases.

S14. MSA of Yaravirus gene 48 and its homologs in mriyaviruses (Mriya_48) and in NCBI Genbank (nr) database.

S15. A, Phylogenetic tree of Mriya_48; B, predicted structures of two Mriya_48 proteins.

S16. MSA of Yaravirus gene 50 and its homologs in mriyaviruses (Mriya_50) and in NCBI Genbank (nr) database.

S17. Predicted structure of Mriya_50 (exemplified with Ple2@KY346835_21). Leucine site chains highlighted in magenta.

S18. MSA of conserved domain of Yaravirus gene 1 and its homologs in mriyaviruses (Mriya_1).

S19. Predicted structure of Mriya_1, exemplified with Ple2@KY346835_29.

S20. A, MSA of Yaravirus gene 51 with mriyavirus and nr homologs. B, Predicted structure of Yaravirus gene 51.

S21. A, Predicted structure of PEV_22; B, same structure overlayed with 6g41_A – the structure of minor capsid protein of Cafeteria mavirus.

S22. Predicted structure of PEV_26 superimposed with gp2.5, a single strand DNA binding protein from bacteriophage T7 (pdb 1je5, green, z-score 13.3).

S23. Predicted structure of Yaravirus minor capsid protein

S24. MSA of Yaravirus minor capsid protein and related sequences.

S1

Yaraviridae MCP

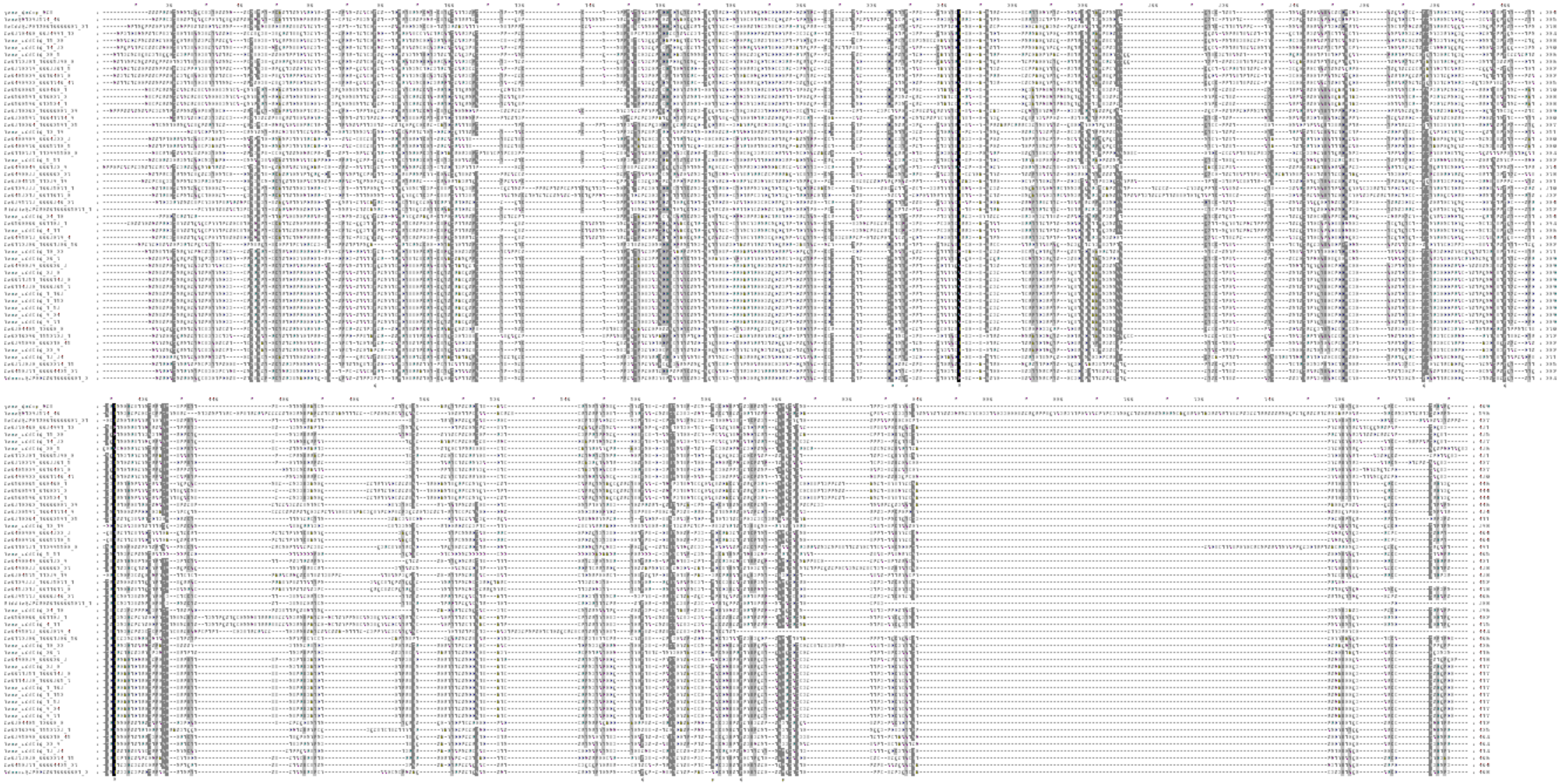

S2

“Gamadviridae” MCP

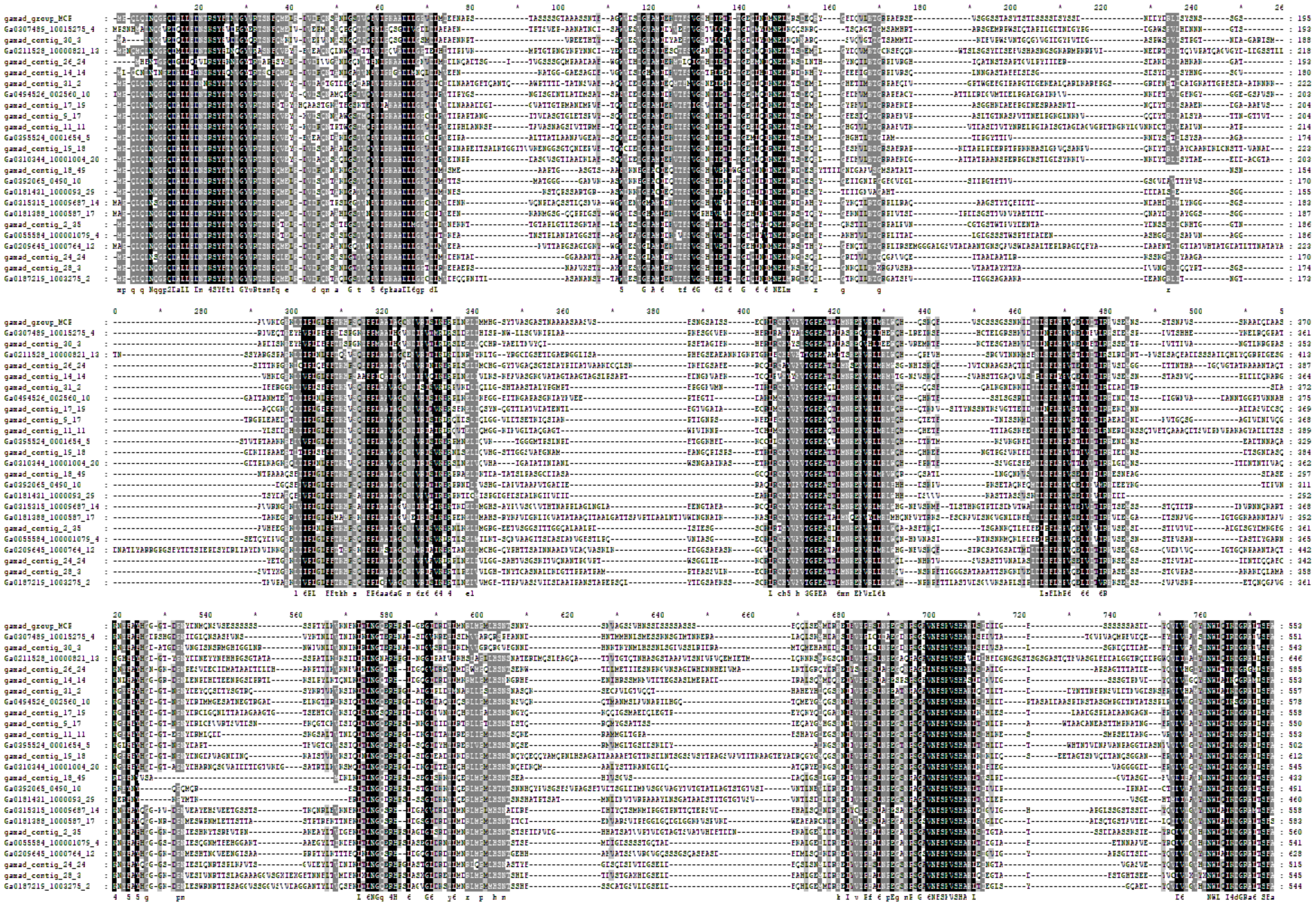

S3

HHpred search initiated from both MCP alignments brings mimi-, irido-, asco-, and phycodnavirus MCP with high confidence.

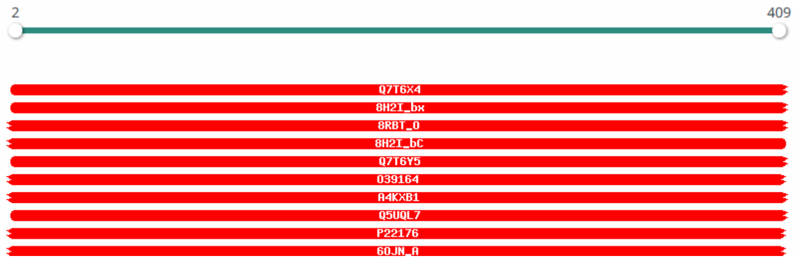

Nr	Hit	Name	Probability	E-value	Score	SS	Aligned cols	Target Length
1	Q7T6X4	CAPS2_MIMIV Probable capsid protein 2 OS=Acanthamoeba polyphaga mimivirus OX=212035 GN=MIMI_R439 PE=3 SV=2	100	2e-74	585.41	34.7	395	591
2	8H2I_bx	MCPv1; giant virus, nucleocytoplasmic large DNA viruses (NCLDVs), viral assembly, Paramecium bursaria chlorella virus 1	100	2.6e-73	573.31	37	397	520
3	8RBT_O	Major capsid protein; cryo-EM, subtomogram averaging, EhV-201, enveloped virus, capsid, major capsid protein, VIRUS; 12.	100	1.3e-72	565.58	39.1	390	496
4	8H2I_bC	MCPv2; giant virus, nucleocytoplasmic large DNA viruses (NCLDVs), viral assembly, Paramecium bursaria chlorella virus 1	100	6.9e-73	560.36	35.9	379	486
5	Q7T6Y5	CAPS4_MIMIV Probable capsid protein 4 OS=Acanthamoeba polyphaga mimivirus OX=212035 GN=MIMI_R441 PE=3 SV=2	100	5.7e-73	567.88	34	391	512
6	O39164	MCP_IRV16 Major capsid protein OS=Costelytra zealandica iridescent virus OX=68348 GN=MCP PE=3 SV=1	100	1.1e-72	563.02	33.3	391	462
7	A4KXB1	CAPSD_HVAVE Major capsid protein OS=Heliothis virescens ascovirus 3e OX=260797 GN=MCP PE=3 SV=1	100	9.5e-73	561.7	32.3	391	455

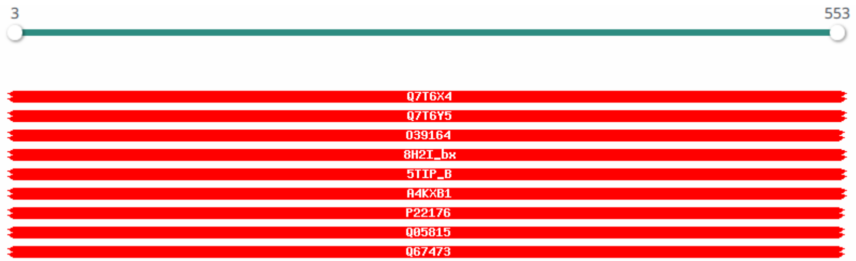

Nr	Hit	Name	Probability	E-value	Score	SS	Aligned cols	Target Length
1	Q7T6X4	CAPS2_MIMIV Probable capsid protein 2 OS=Acanthamoeba polyphaga mimivirus OX=212035 GN=MIMI_R439 PE=3 SV=2	100	1.3e-77	669.27	40.4	419	591
2	Q7T6Y5	CAPS4_MIMIV Probable capsid protein 4 OS=Acanthamoeba polyphaga mimivirus OX=212035 GN=MIMI_R441 PE=3 SV=2	100	1.9e-76	651.33	44.5	425	512
3	O39164	MCP_IRV16 Major capsid protein OS=Costelytra zealandica iridescent virus OX=68348 GN=MCP PE=3 SV=1	100	3e-76	643.72	44.8	434	462
4	8H2I_bx	MCPv1; giant virus, nucleocytoplasmic large DNA viruses (NCLDVs), viral assembly, Paramecium bursaria chlorella virus 1	100	5.4e-76	650.01	45.8	408	520
5	5TIP_B	Major capsid protein; VIRAL PROTEIN; HET: 7CV, XYP, FUC, BGC, RM4, MAN, XXR, GLA; 2.0A {Paramecium bursaria Chlorella vi	100	7.3e-76	636.15	42.6	378	436
6	A4KXB1	CAPSD_HVAVE Major capsid protein OS=Heliothis virescens ascovirus 3e OX=260797 GN=MCP PE=3 SV=1	100	1.4e-75	637.32	44.6	411	455
7	P22176	MCP_LCDV1 Major capsid protein OS=Lymphocystis disease virus 1 (isolate Darai) OX=654922 GN=MCP PE=3 SV=2	100	9.2e-76	639.42	43.3	404	459

top HHpred hits for Yaraviridae (left) and “Gamadviridae” (right) MCP alignments

S4

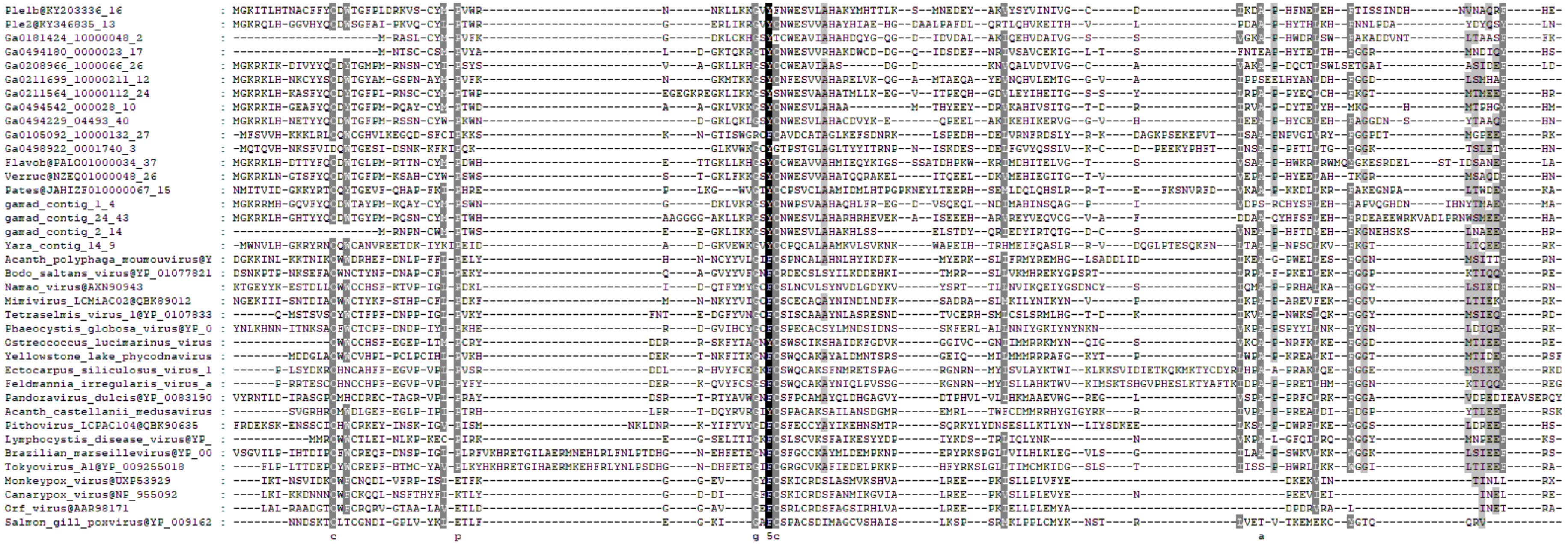
Ple1b@KY203336_16
Ple2@KY346835_13
Ga0181424_10000048_2
Ga0494180_0000023_17
Ga0208966_1000066_26
Ga0211699_10000211_12
Ga0211564_10000112_24
Ga0494542_000028_10
Ga0494229_04493_40
Ga0105092_10000132_27
Ga0498922_0001740_3
Flavob@PALC01000034_37
Verruc@NZEQ01000048_26
Pates@JAHIZF010000067_15
gamad_contig_1_4
gamad_contig_24_43
gamad_contig_2_14
Yara_contig_14_9
Acanth_polyphaga_moumouvirus@Y
Bodo_saltans_virus@YP_01077821
Namao_virus@AXN90943
Mimivirus_LCMiAC02@QBK89012
Tetraselmis_virus_1@YP_0107833
Phaeocystis_globosa_virus@YP_0
Ostreococcus_lucimarinus_virus
Yellowstone_lake_phycodnavirus
Ectocarpus_siliculosus_virus_1
Feldmannia_irregularis_virus_a
Pandoravirus_dulcis@YP_0083190
Acanth_castellanii_medusavirus
Pithovirus_LCPAC104@QBK90635
Lymphocystis_disease_virus@YP_
Brazilian_marseillevirus@YP_00
Tokyovirus_A1@YP_009255018
Monkeypox_virus@UXP53929
Canarypox_virus@NP_955092
Orf_virus@AAR98171
Salmon_gill_poxvirus@YP_009162

S5

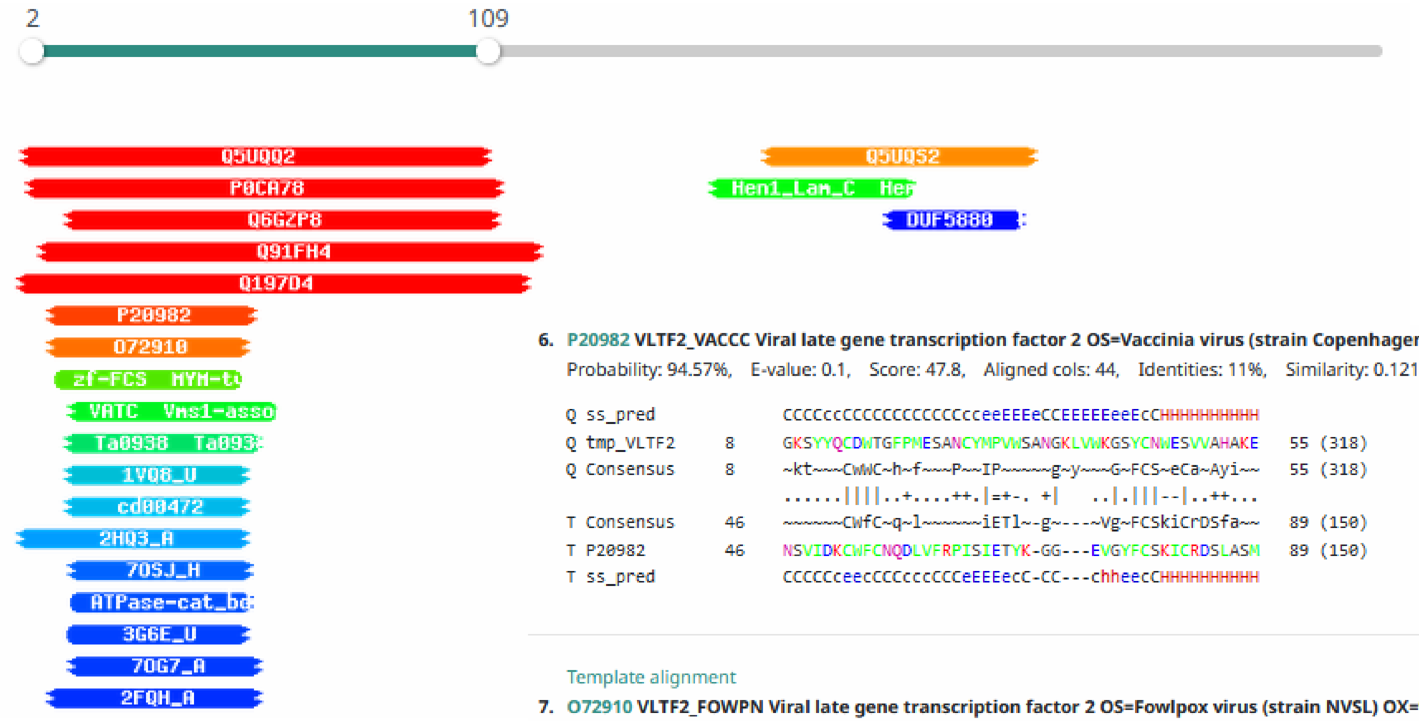

6. **P20982 VLTF2_VACCC Viral late gene transcription factor 2 OS=Vaccinia virus (strain Copenhagen) OX=10249 GN=VLTF2 PE=2 SV=1**

Probability: 94.57%, E-value: 0.1, Score: 47.8, Aligned cols: 44, Identities: 11%, Similarity: 0.121, Template Neff: 3.5

```
Q ss_pred                CCCCccCCCCCCCCCCCCccceeEEEeCCEEEEEeeEcCHHHHHHHHHH
Q tmp_VLTF2       8      GKSYYQCDWTGFPMESANCYMPVWSANGKLVWKGSYCNWESVVAHAKE   55 (318)
Q Consensus       8      ~kt~~~CWWC~h~f~~~~P~~IP~~~~~~g~y~~~~G~FCS~eCa~Ayi~~   55 (318)
                         ......||||..+....++.|=+-. +|    ..|.|||--|..++...
T Consensus      46      ~~~~~~~~CWfC~q~l~~~~~~~~iETl~-g~---~Vg~FCSkiCrDSfa~~   89 (150)
T P20982         46      NSVIDKCWFCNQDLVFRPISIETYK-GG---EVGYFCSKICRDSLASM   89 (150)
T ss_pred                 CCCCCceecCCCccccCCCeEEEEecC-CC---chheecCHHHHHHHHHH
```

Template alignment

7. **O72910 VLTF2_FOWPN Viral late gene transcription factor 2 OS=Fowlpox virus (strain NVSL) OX=928301 GN=VLTF2 PE=2 SV=1**

Probability: 90.65%, E-value: 0.75, Score: 42.63, Aligned cols: 42, Identities: 14%, Similarity: 0.174, Template Neff: 3.4

```
Q ss_pred                CCCCccCCCCCCCCC-CCCCcceeEEEeCCEEEEEeeEcCHHHHHHHH
Q tmp_VLTF2       8      GKSYYQCDWTGFPM-ESANCYMPVWSANGKLVWKGSYCNWESVVAHA   53 (318)
Q Consensus       8      ~kt~~~CWWC~h~f-~~~~P~~IP~~~~~~g~y~~~~G~FCS~eCa~Ayi   53 (318)
                         ......||+|.... ...++.|=+-. +|    ..|.|||-=|..+++
T Consensus      46      ~~~~~~~~CWfC~q~i~~~~~~~~~~iETl~-G~---~IG~FCS~ICrDSfA   88 (154)
T O72910         46      RKDKDNCWFCKQDMNTYNPYFIETLY-GD---HIGVFCSKICRDSFA   88 (154)
T ss_pred                 cCCCCceeccCCccccCCCCeEEEEec-cc---cceeeehHHhHHHHH
```

S6

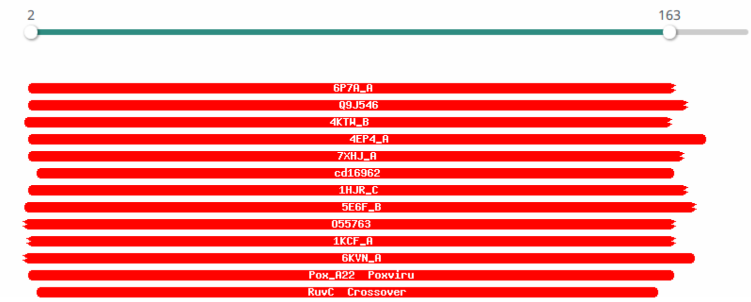

Nr	Hit	Name	Probability	E-value	Score	SS	Aligned cols	Target Length
1	6P7A_A	Holliday junction resolvase; RESOLVASE, HYDROLASE; 3.081A {Fowlpox virus}	99.78	4.2e-17	115.02	14.9	138	148
2	Q9J546	RUVV_FOWPN Holliday junction resolvase OS=Fowlpox virus (strain NVSL) OX=928301 GN=FPV187 PE=1 SV=1	99.78	2.2e-17	117.98	13.3	145	156
3	4KTW_B	RuvC endonuclease; endonuclease, DNA junctions, replication, recombination, phage packaging, Holliday junction, RNase-H	99.77	5.9e-17	116.12	15.1	141	161
4	4EP4_A	Crossover junction endodeoxyribonuclease RuvC; resolvase, Hydrolase; 1.28A {Thermus thermophilus} SCOP: c.55.3.6	99.75	1.4e-16	114.2	14.3	148	166
5	7XHJ_A	Crossover junction endodeoxyribonuclease RuvC; nuclease, DNA repair, Homologous recombination, Holliday junction resolva	99.74	3e-16	114.34	13.9	140	179
6	cd16962	RuvC; Crossover junction endodeoxyribonuclease RuvC. Crossover junction endodeoxyribonuclease RuvC is also called Hollid	99.73	4e-16	110.27	14.1	138	156
7	1HJR_C	HOLLIDAY JUNCTION RESOLVASE (RUVC); SITE-SPECIFIC RECOMBINASE; 2.5A {Escherichia coli} SCOP: c.55.3.6	99.73	2.6e-16	111.81	12.8	141	158
8	5E6F_B	CNPV261 Holliday junction resolvase protein; Poxvirus, Resolvase, Holliday Junction, Metal-binding, VIRAL PROTEIN; HET:	99.73	1.7e-15	107.76	16.7	146	152

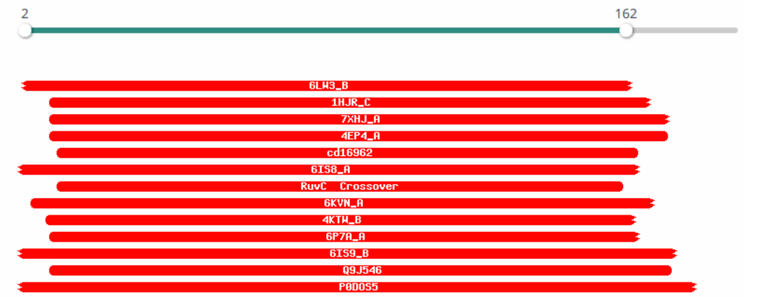

Nr	Hit	Name	Probability	E-value	Score	SS	Aligned cols	Target Length
1	6LW3_B	Crossover junction endodeoxyribonuclease RuvC; Holliday junction resolvase RuvC Endonuclease, DNA BINDING PROTEIN; 2.38A	99.68	1.4e-14	101.5	16	147	163
2	1HJR_C	HOLLIDAY JUNCTION RESOLVASE (RUVC); SITE-SPECIFIC RECOMBINASE; 2.5A {Escherichia coli} SCOP: c.55.3.6	99.68	1.4e-14	101.01	15.7	145	158
3	7XHJ_A	Crossover junction endodeoxyribonuclease RuvC; nuclease, DNA repair, Homologous recombination, Holliday junction resolva	99.67	1.9e-14	102.89	16	151	179
4	4EP4_A	Crossover junction endodeoxyribonuclease RuvC; resolvase, Hydrolase; 1.28A {Thermus thermophilus} SCOP: c.55.3.6	99.65	7.1e-14	98.46	16.8	152	166
5	cd16962	RuvC; Crossover junction endodeoxyribonuclease RuvC. Crossover junction endodeoxyribonuclease RuvC is also called Hollid	99.65	5e-14	97.77	15.4	142	156
6	6IS8_A	Monokaryotic chloroplast 1; Holliday junction resolvase-DNA complex, DNA BINDING PROTEIN-DNA complex; HET: 1PE; 1.68A {Z	99.61	1.1e-13	98.5	14.5	151	174
7	PF02075.21	; RuvC ; Crossover junction endodeoxyribonuclease RuvC	99.6	1.8e-13	94.57	14.4	137	148
8	6KVN_A	NtMOC1; chloroplast, resolvase, PLANT PROTEIN; 2.02A {Nicotiana tabacum}	99.6	2.7e-13	96.23	15.3	150	171

top HHpred hits for “Gamadviridae” (right) and Yaraviridae (left) RuvC alignments

S7

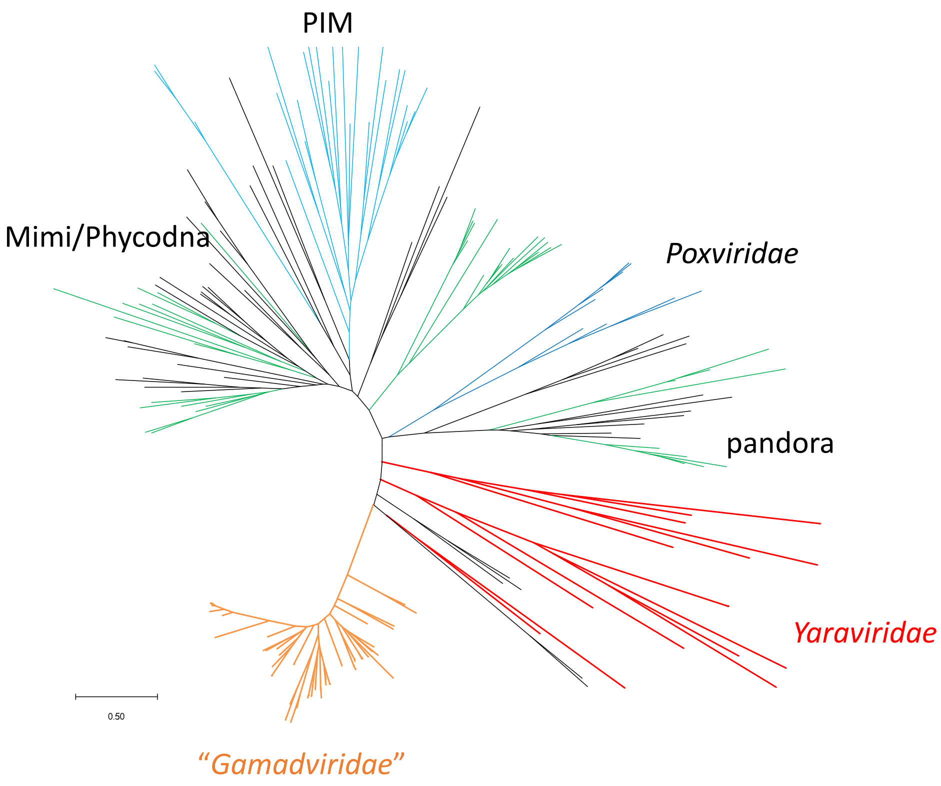

PIM
Mimi/Phycodna
Poxviridae
pandora
Yaraviridae
"Gamadviridae"
0.50

S8

SF3_hel1 (most of *Yaraviridae*; Yaravirus gene 69)

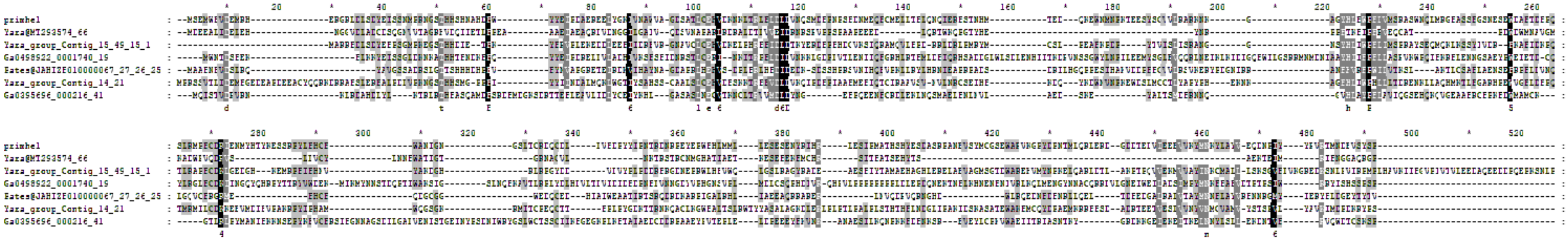

primase domain

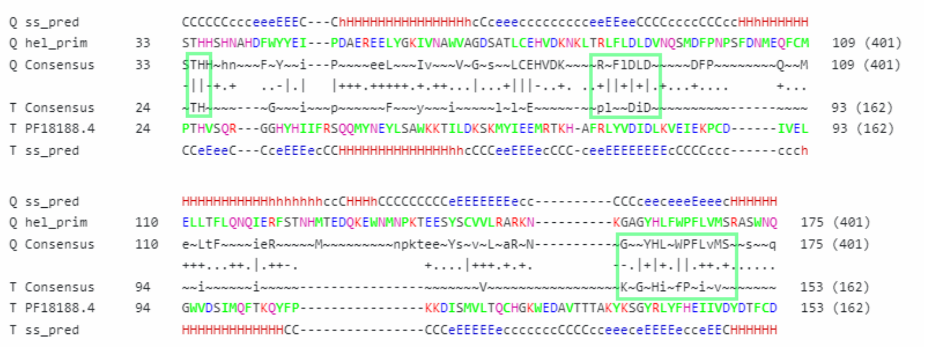

helicase domain

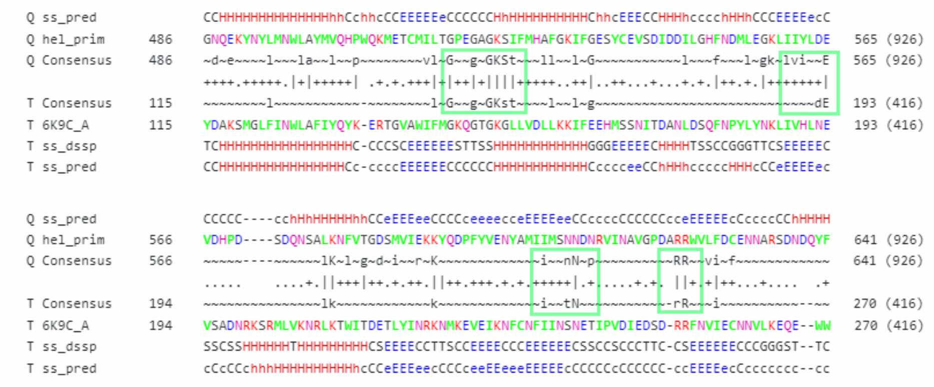

SF3_hel1 (Yaravirus gene 69)

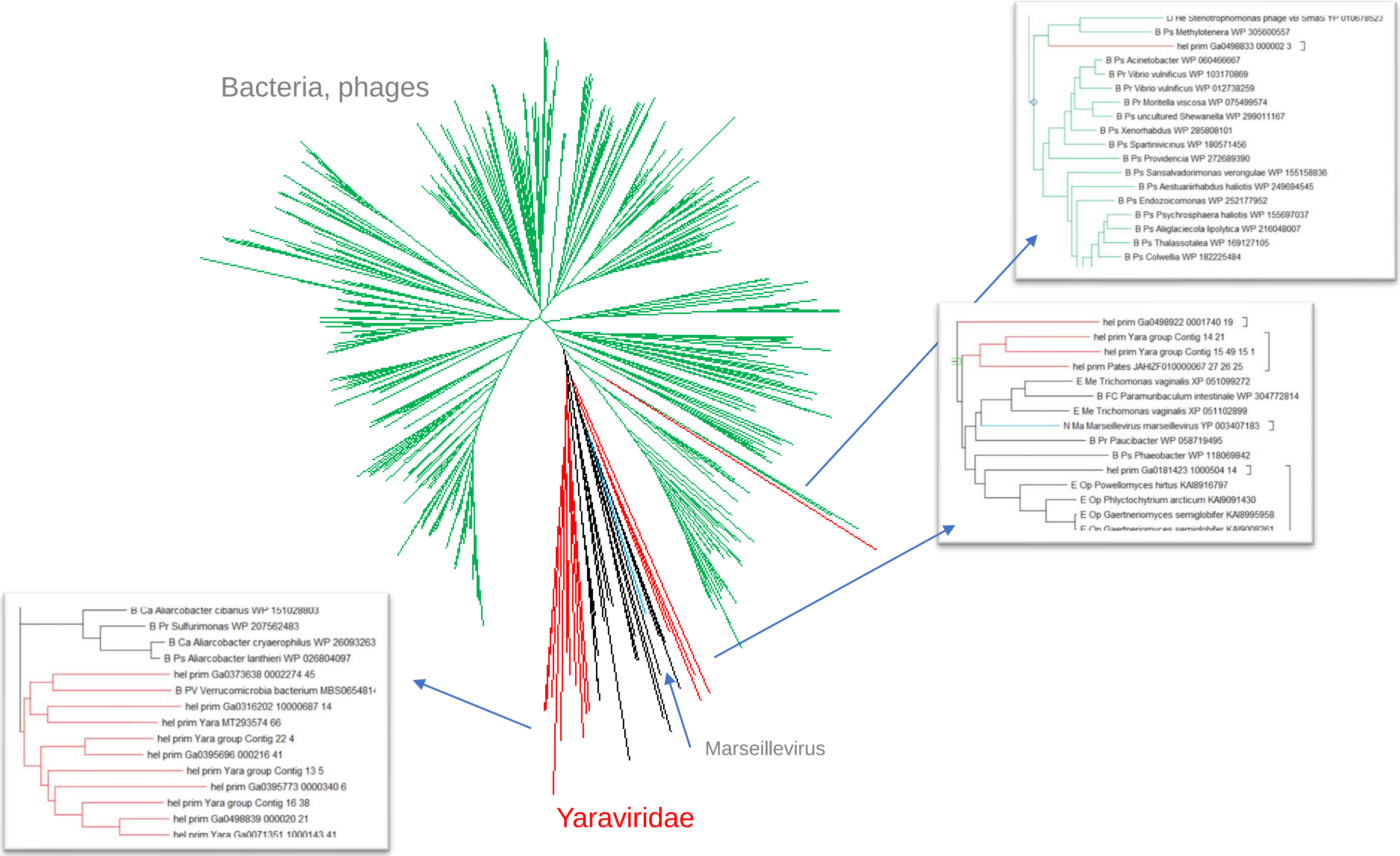

S10

SF3_hel2

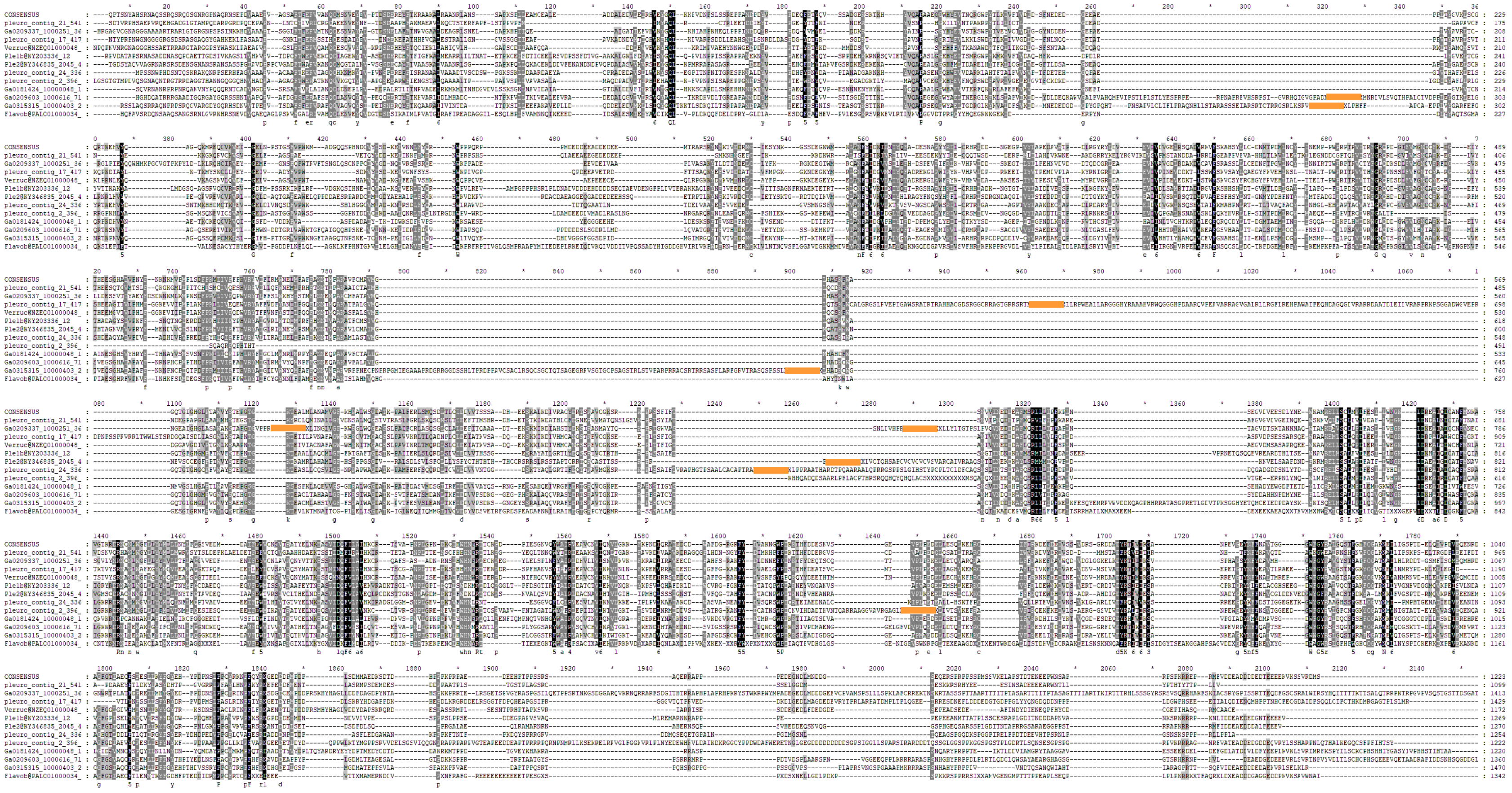

some SF3_hel2 proteins have frameshifts (marked in orange on the figure).

S11

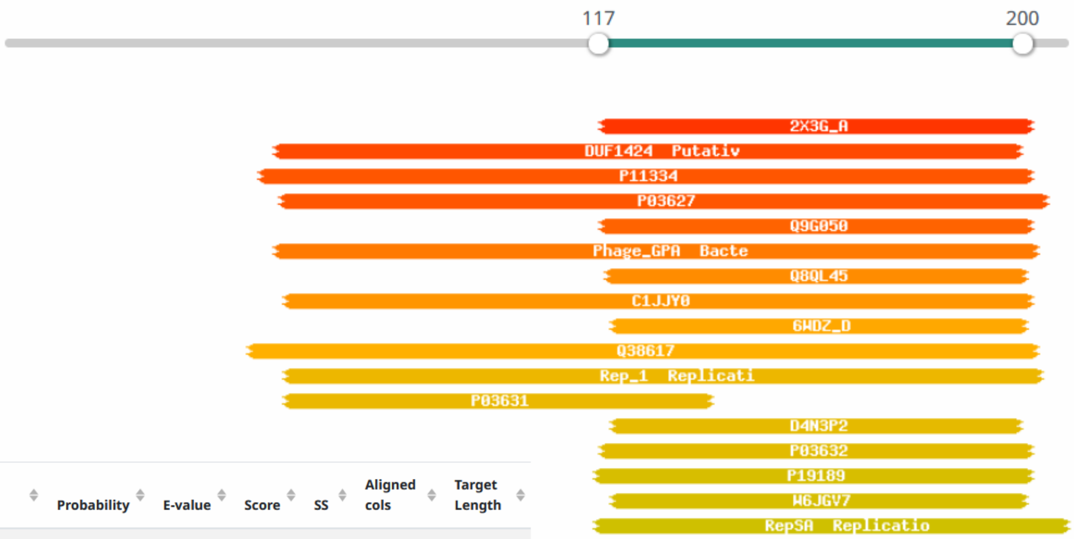

Nr	Hit	Name	Probability	E-value	Score	SS	Aligned cols	Target Length
1	2X3G_A	SIRV1 HYPOTHETICAL PROTEIN ORF119; VIRAL PROTEIN, HOST MEMBRANE; HET: MSE; 1.8A {SULFOLOBUS ISLANDICUS RUDIVIRUS 1 VARIA	95.52	0.097	42.87	6	65	119
2	PF07232.14	; DUF1424 ; Putative rep protein (DUF1424)	93.77	6.8	36.39	13.8	115	316
3	P11334	REP_SPV4 Replication-associated protein ORF2 OS=Spiroplasma virus 4 OX=10855 GN=ORF2 PE=3 SV=1	92.74	3.1	39.96	10.4	107	320
4	P03627	REP_BPPF3 Replication-associated protein G2P OS=Pseudomonas phage Pf3 OX=10872 GN=II PE=3 SV=1	92.66	8.1	36.1	12.6	109	278
5	Q9G050	REP_BPPHM Replication-associated protein VP4 OS=Bdellovibrio phage phiMH2K OX=145579 GN=ORF4 PE=3 SV=1	91.61	1.4	42.09	6.8	67	315
6	PF05840.16	; Phage_GPA ; Bacteriophage replication gene A protein (GPA)	89.69	12	35.26	10.7	119	316
7	Q8QL45	Y117_SIRV1 Uncharacterized protein 117 OS=Sulfolobus islandicus rod-shaped virus 1 OX=157898 GN=117 PE=4 SV=1	88.17	2.8	35.65	5.1	63	117

S12

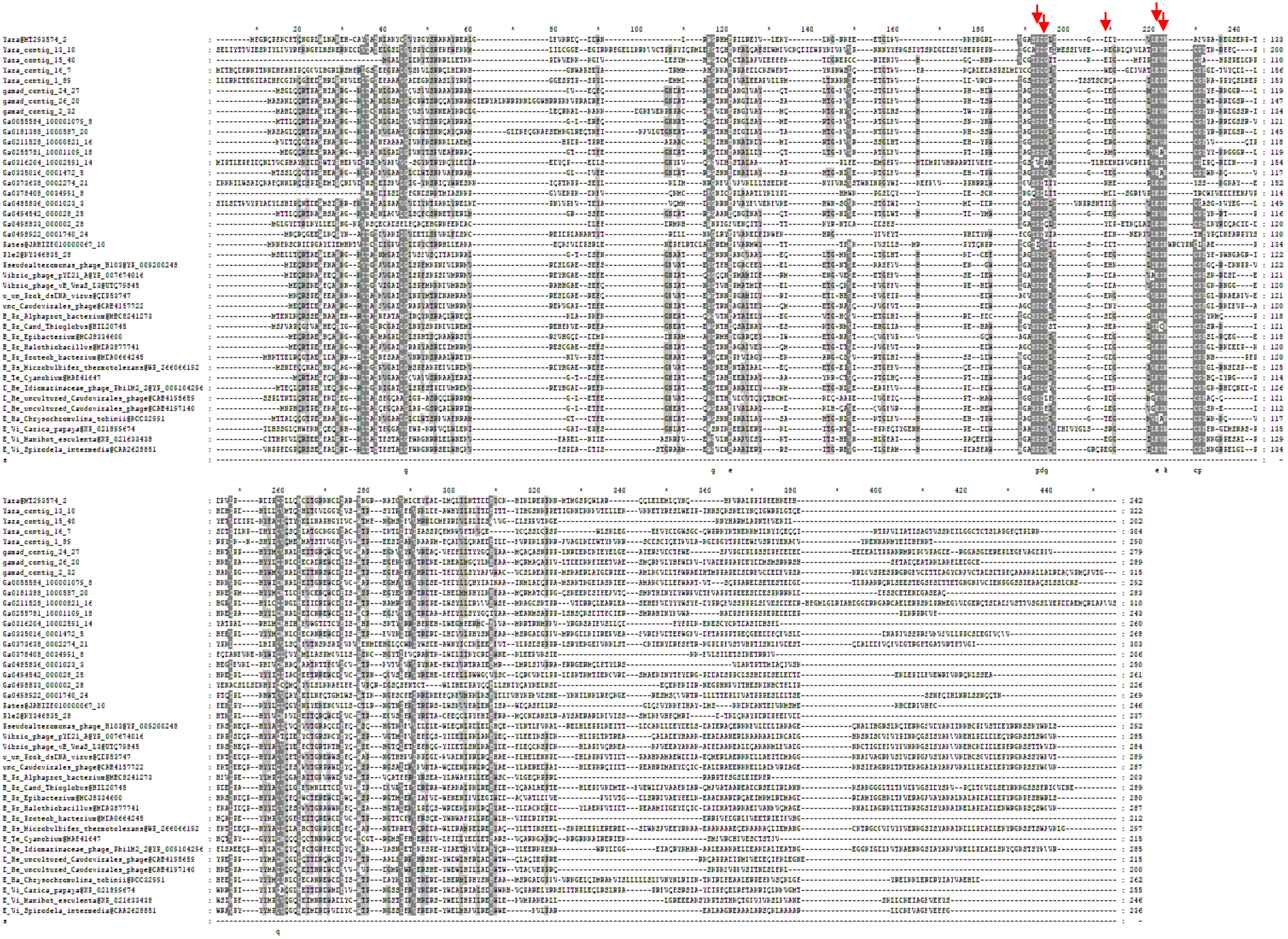

S13

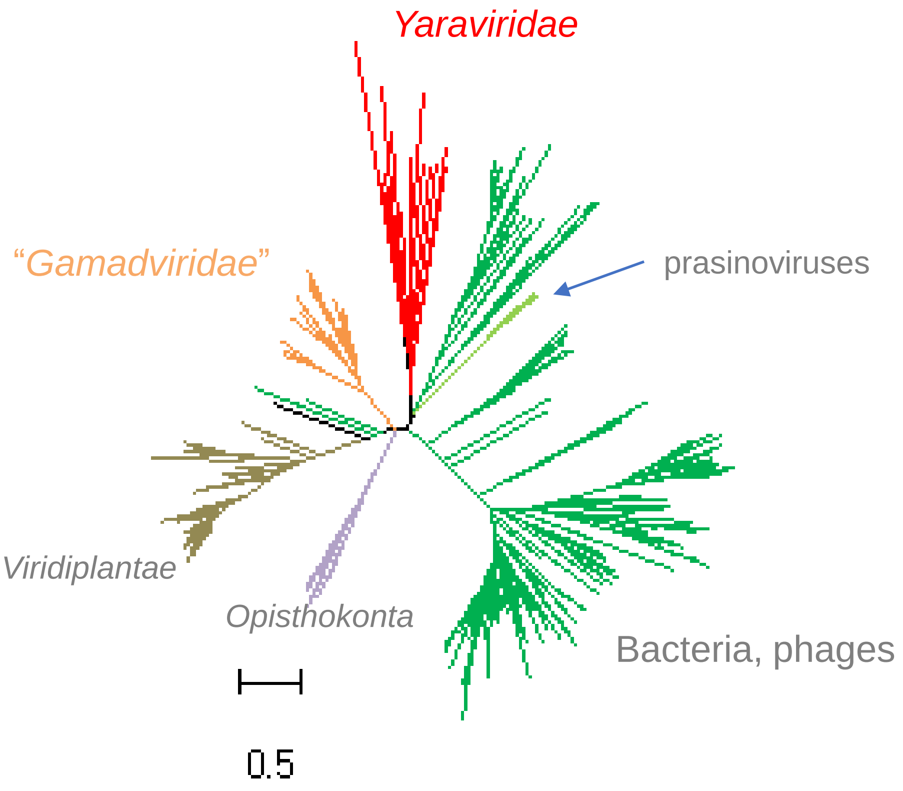

Yaraviridae
"Gamadviridae"
prasinoviruses
Viridiplantae
Opisthokonta
Bacteria, phages
0.5

S14

gene48 with nr hits

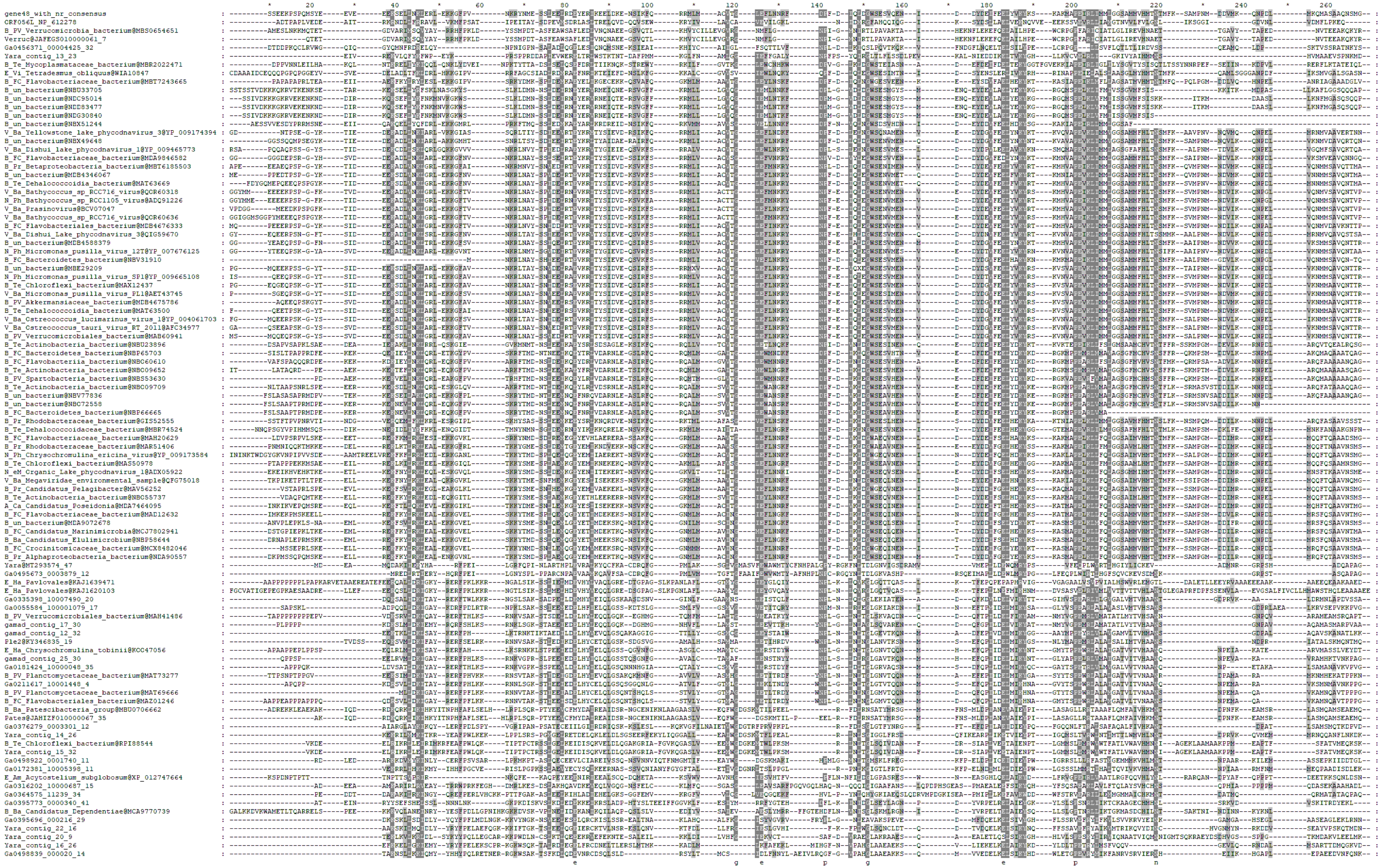

S15

A

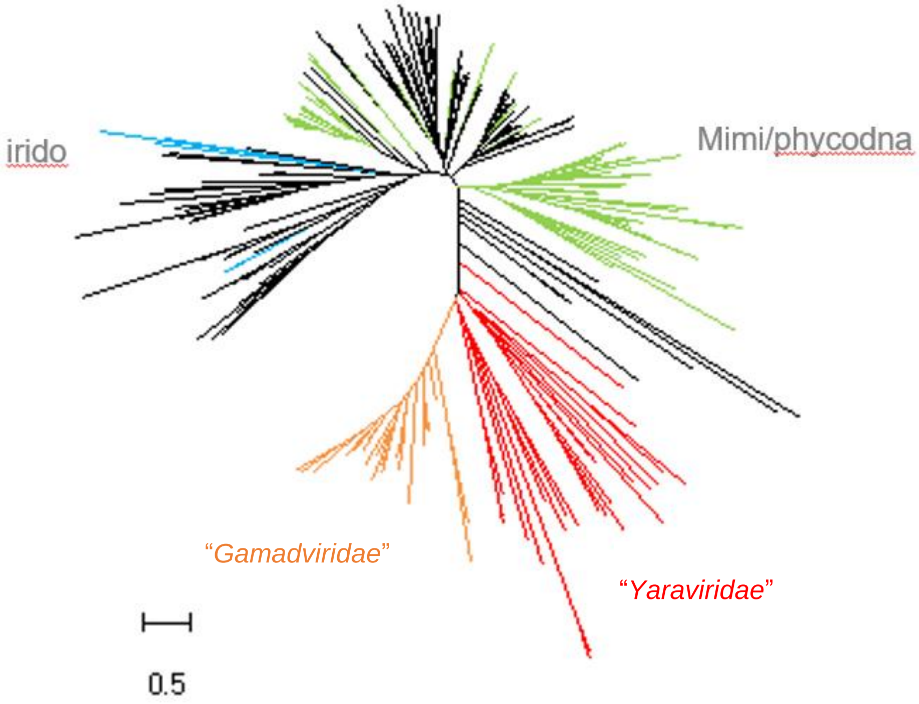

B

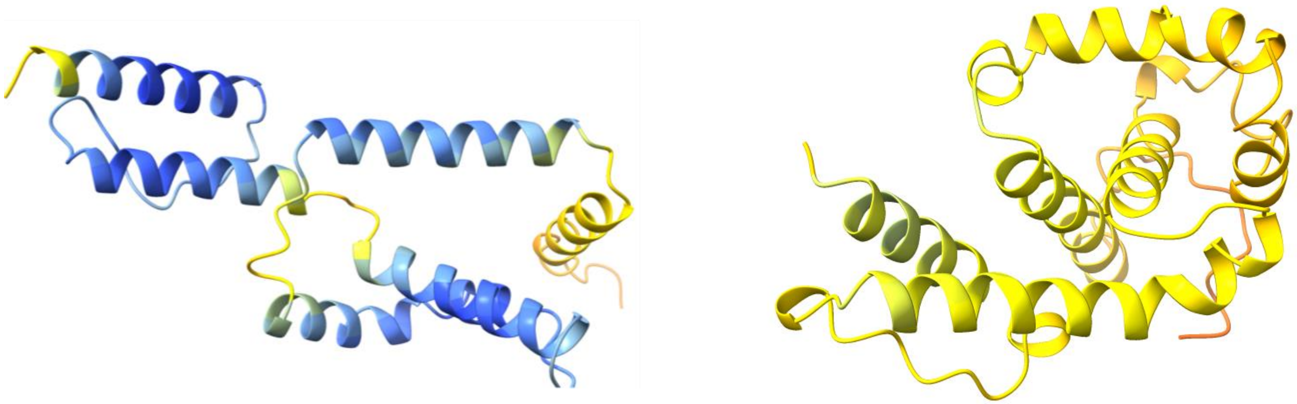

gene48 (Ple2@KY346835_19)

(Yaravirus)

S16

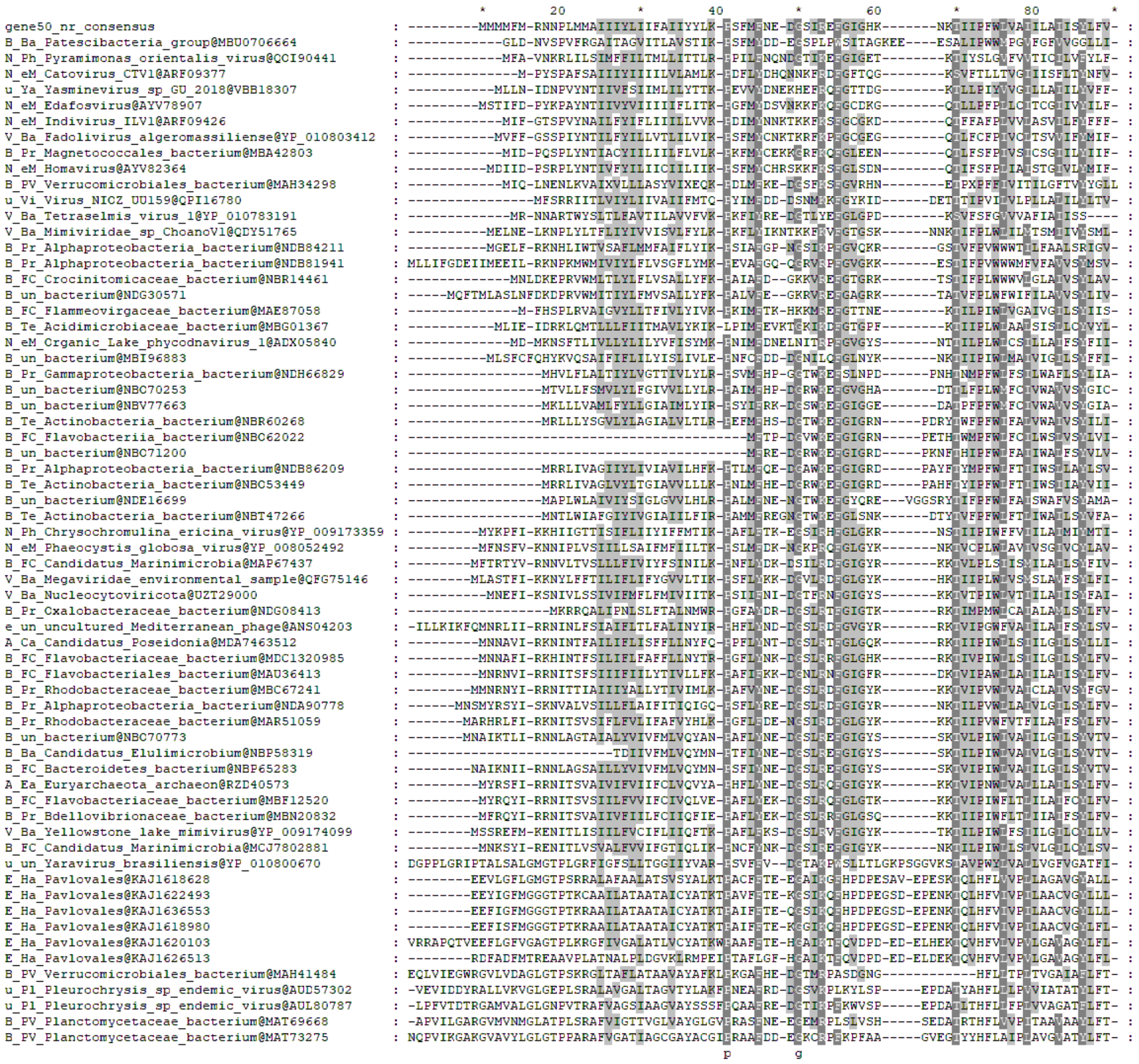

Mriya_50 with nr hits

S17

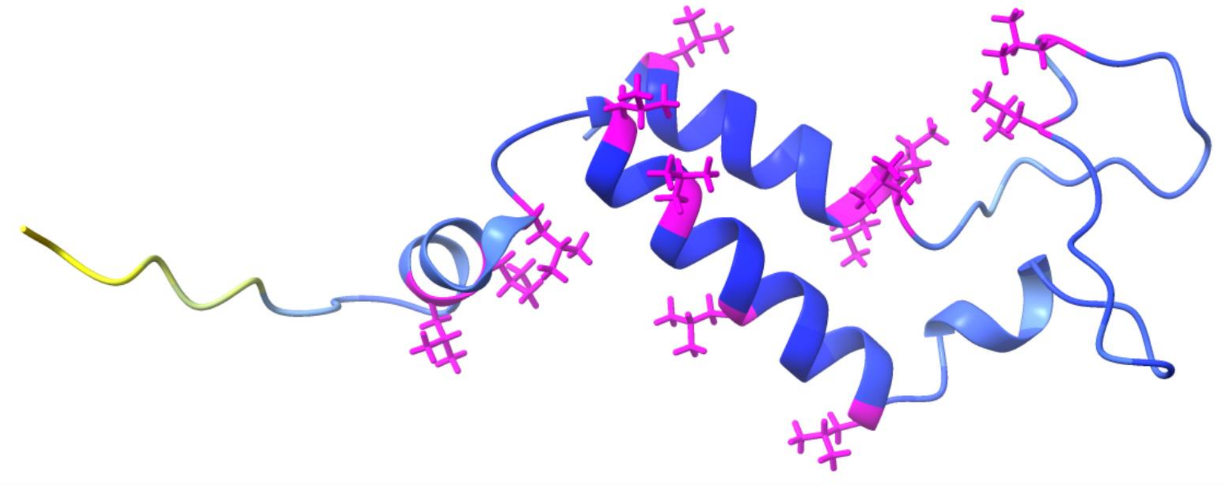

Mriya_50 (Ple2@KY346835_21)

S18

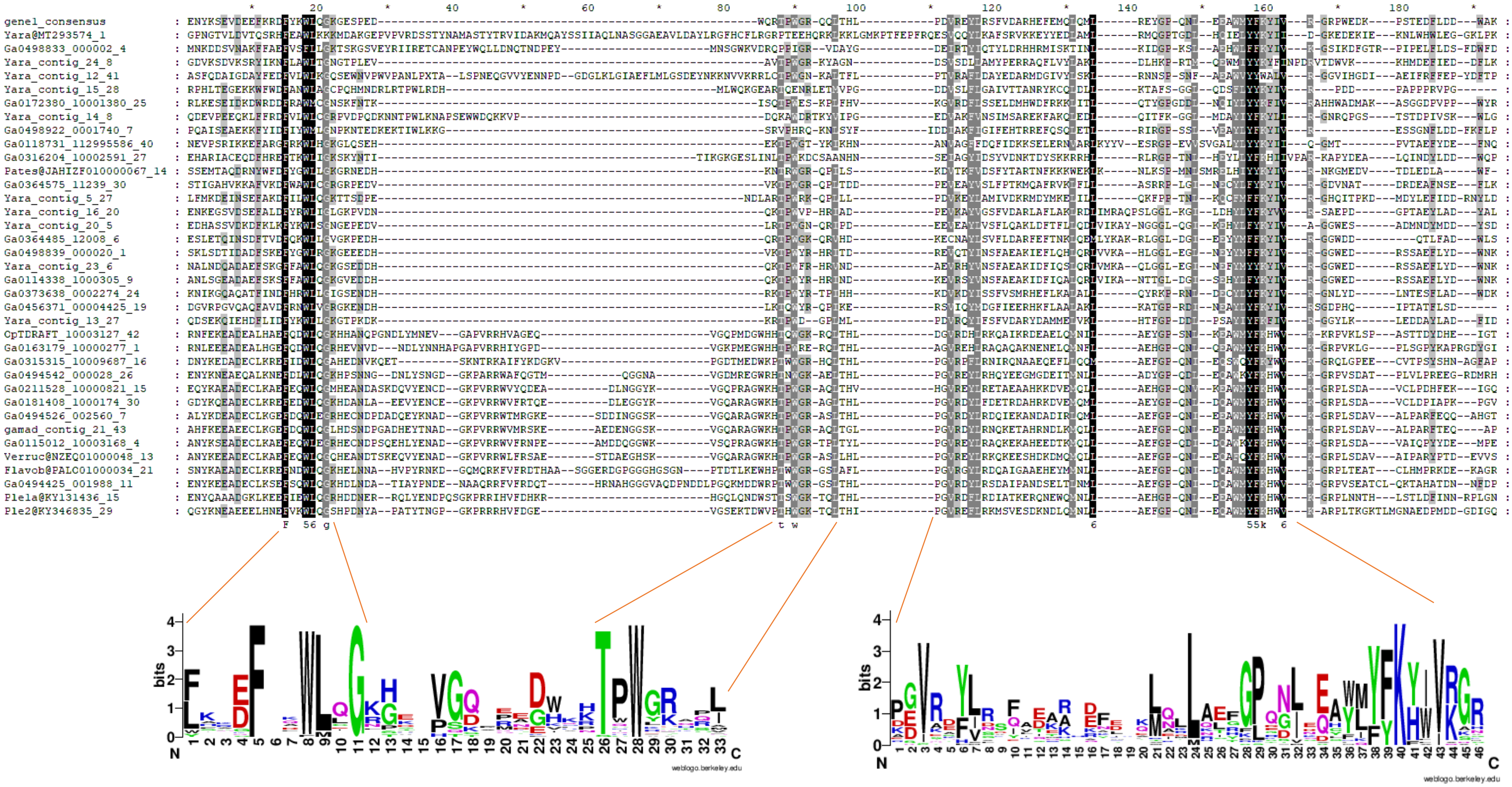

S19

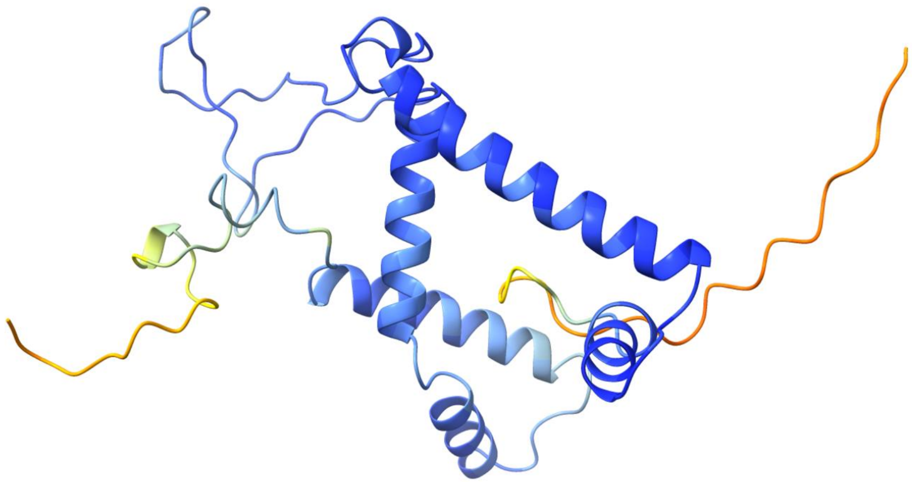

Mriya_1, exemplified with Ple2@KY346835_29

Mriya_51

S20

A

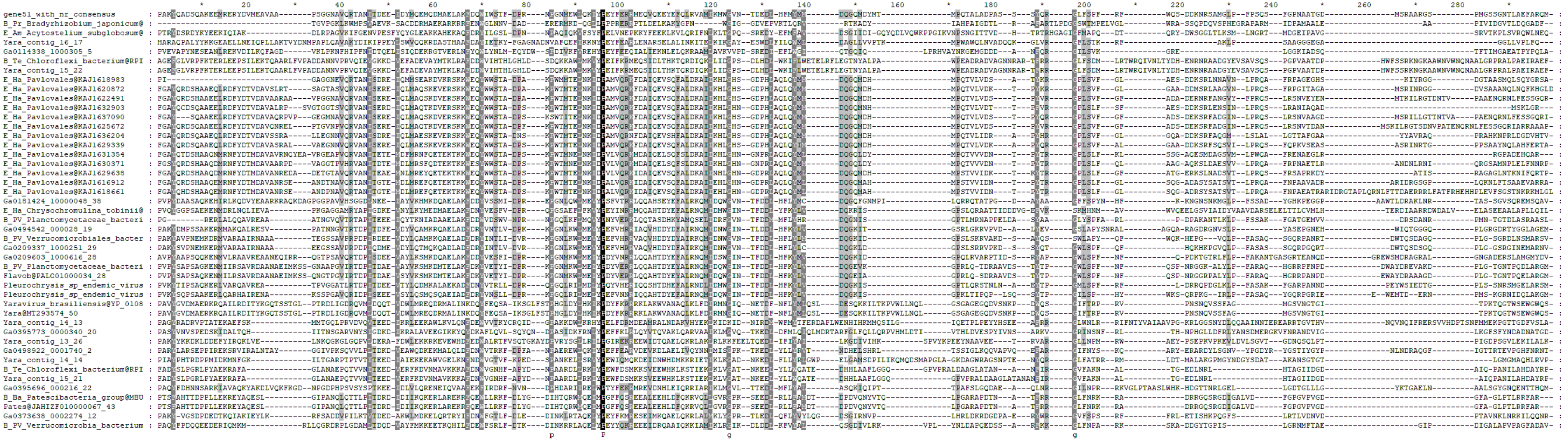

B

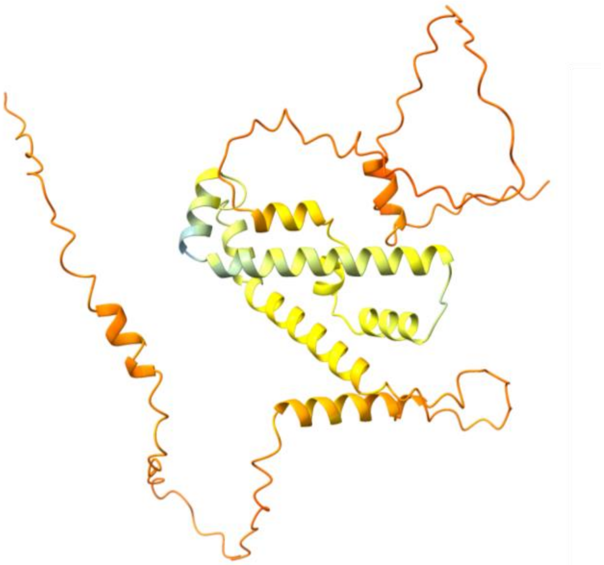

(Yara@MT293574_50, aka YP_010800671)

S21

A

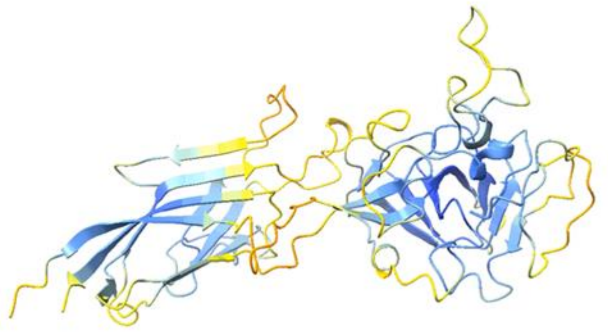

Foldseek:
4 uncharacterized proteins

Dali
6.4 , 6g41-A , MOLECULE: MINOR CAPSID PROTEIN;
→ Cafeteriavirus-dependent mavirus (Varidnaviria, Bamfordvirae, Preplasmiviricota)
5.4 , 3j26-N , MOLECULE: CAPSID PROTEIN V20;
→ resolution structure of the Sputnik virophage by cryo-EM

B

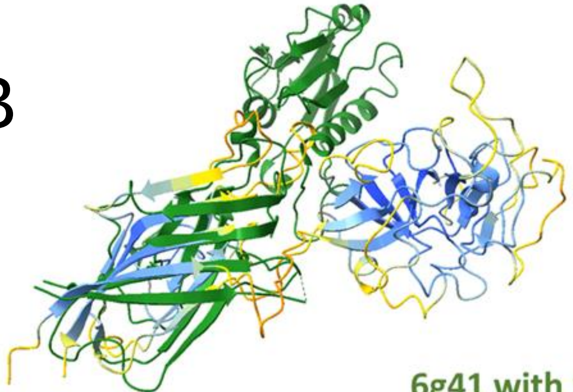

6g41 with SJR (overlayed with Ple_22)
and insertion domain

S22

PEV_26 - ssb

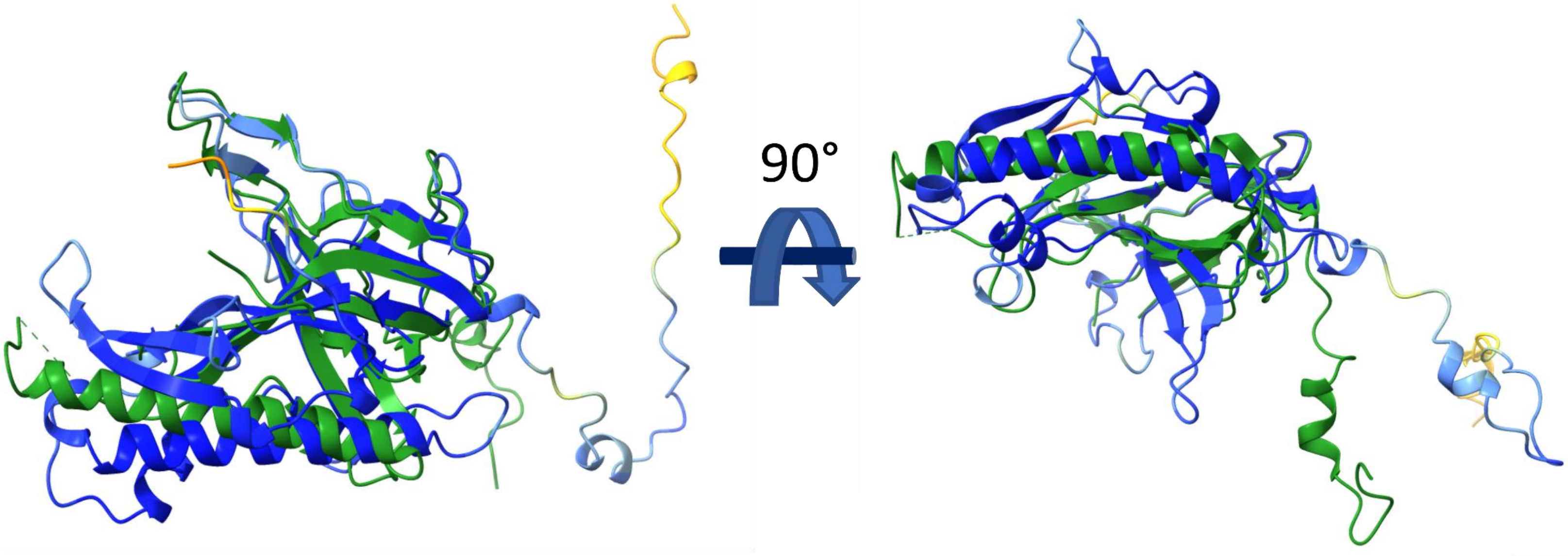

S23

Yaravirus minor capsid protein (YP_010800666.1)

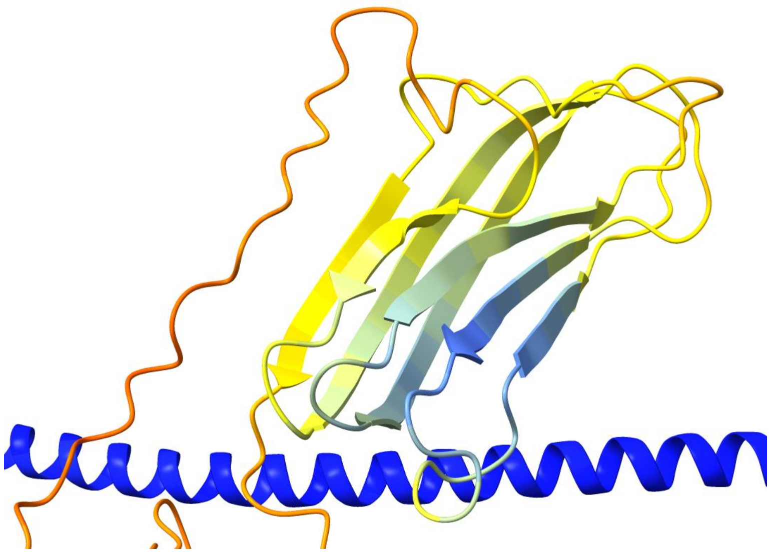

S24

Yaravirus minor capsid protein (C-terminal of YP_010800666.1)

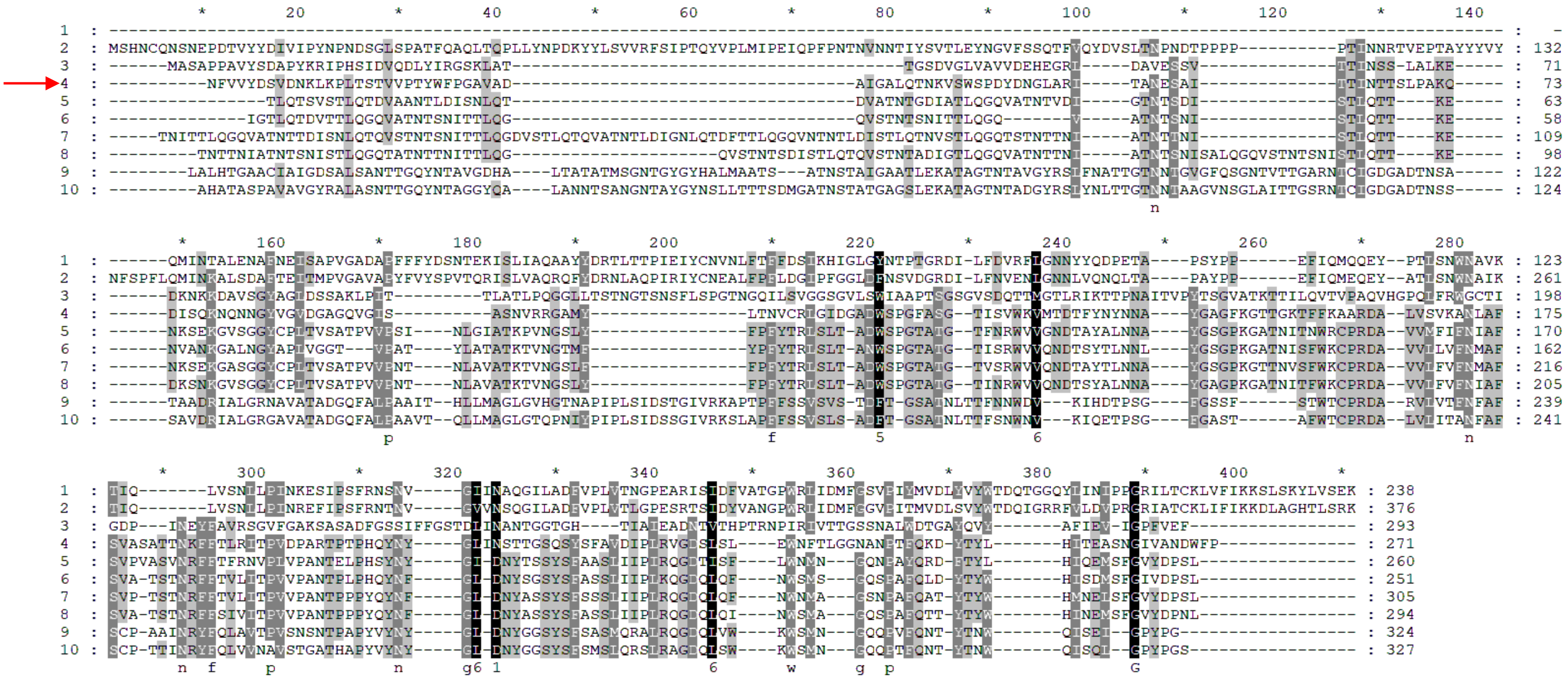

1 Sputnik_UMZ08530 .1 minor virion protein [Mimivirus-dependent virus Sputnik]
2 Zamilon_YP_008859634 .1 minor virion protein [Zamilon virus]
3 YP_010800667.1 hypothetical protein QKS73_gp57 [Yaravirus brasiliensis]
4 YP_010800666.1:200-470 tail needle DNA stabilization protein [Yaravirus brasiliensis]
5 YP_009507121.1:265-524 chromosome segregation protein (SMC) [Tunisvirus fontaine2]
6 YP_009094634.1:305-555 hypothetical protein MEL_133 [Melbournevirus]
7 YP_009345180.1:241-545 hypothetical protein B1750_gp035 [Noumeavirus]
8 YP_009238991.1:321-614 hypothetical protein A3303_gp486 [Brazilian marseillevirus]
9 YP_010804828.1:961-1284 hypothetical protein QKU73_gp304 [Acanthamoeba castellanii medusavirus]
10 YP_010802084.1:451-777 hypothetical protein QKT49_gp315 [Acanthamoeba castellanii medusavirus]
